# Effect of Predry-treatment on the bioactive constituents and quality of avocado (*Persea americana* Mill.) oil from three cultivars growing in China

**DOI:** 10.3389/fnut.2023.1230204

**Published:** 2023-07-17

**Authors:** Jiashui Wang, Hongbin Yang, Peicong Wu, Jiali Zhang, Weihong Ma, Yanxia Li, Jinping Liu

**Affiliations:** ^1^Tropical Crops Genetic Resources Institute, Haikou Experimental Station, Key Laboratory of Biology and Genetic Resources of Tropical Crops, Chinese Academy of Tropical Agricultural Sciences, Haikou, China; ^2^College of Agriculture, Hainan University, Haikou, China

**Keywords:** avocado oil, predry-treatment, avocado cultivar, bioactive constituents, quality assessment

## Abstract

Avocado oil has gained a lot of favor in foods and cosmetics because of its high-quality fatty acid composition and bioactive components. This study aimed to compare the effect of various predry-treatments on the yield and quality of avocado oil from three Chinese avocado (*Persea americana* Mill.) varieties (*Hass, Reed*, and *Pinkerton*). The results showed that drying methods had significant effect on the avocado oil yield and its composition. Among the three drying methods the highest yield was obtained by freeze drying, and *Hass* showed the highest yield in the three avocado varieties with its oil owning the lowest peroxide and anisidine value. *Reed* oil owned the highest levels of functional micronutrients (e.g., tocopherols, phenolics, squalene). Vacuum drying resulted in higher concentrations of tocopherols, phytosterols, phenolics, squalene, and thus rendered greater DPPH and ABTS scavenging activity. These results are important to improve the quality of Chinese avocado oil.

## 1. Introduction

Avocado (*Persea Americana* Mill.) is nutritious fruit widely distributed in tropical and subtropical regions. Its cultivation and processing are gaining popularity due to the multi-faceted salutary benefits associated with its consumption. The last several years have witnessed the growing demand for its oil because of its great potential applications in human nutrition, cosmetic and pharmaceutical industries (1–3). One remarkable fact about avocado oil (AO) is that it contains essential fatty acids (~13%), such as linoleic and linolenic acid, and monounsaturated fatty acids (~60%) that are salutary to the human cardiovascular system (2, 4). Moreover, AO has a higher phenolic content than other tropical and subtropical oils, and its high antioxidant, antibacterial properties and other health-promoting effects, such as lower risk of cataracts, diabetes, coronary heart disease, chemoprevention, age-related macular disease and prostate cancer, have been reported (1, 5).

Interest in developing and improving extraction technology that preserves the nutritional and physicochemical characteristics of AO as much as possible has been increasing. Many factors, including climatic conditions, growing region, variety and degree of fruit ripeness, can influence the chemical composition of AO, and extraction as well as processing techniques have been reported to significantly influence the yield and fatty acid composition of AO from Chile, Peru and Spain varieties (6, 7). With the large-scale cultivation of avocado fruit in China (e.g., Hainan, Guangxi, Yunnan and etc.), the development of avocado varieties with high-quality AO has attracted more and more attention. *Hass*, *Reed*, and *Pinkerton* are the three most profitable avocado varieties in China, which represent as many as two-thirds of the domestic avocado production. Moreover, extraction of AO is significantly affected by the drying of avocado pulp as it affects the physiochemical properties of oil (2, 8, 9). Mostly in modern industry, AO is extracted through vacuum drying in low temperature (10) or air drying with microwaves (8, 11, 12). However, these methods only slightly improve the extraction and produce AO with little quality improvement compared to those obtained by conventional drying methods such as oven drying with forced air circulation as well as traditional solvent extraction techniques. Additionally, oil quality is impacted by the oil composition *per se* and could be influenced by many processing factors like pulp drying (7, 13). However, there is little investigation on the influence of Chinese avocado varieties and avocado fruit processing (for instance pulp drying) on the quality of AO.

Therefore, the objective of this study was to evaluate the quality of AO extracted from three avocado cultivars (*Hass*, *Reed*, and *Pinkerton*) growing in China whose pulp was subjected to three drying pretreatments (freeze drying, vacuum drying and oven drying). The impact of drying treatments on the extraction efficiency and quality of AO were investigated. Moreover, the physicochemical properties and the functional components of AO were systematically analyzed. The combination of drying pretreatment and avocado variety in China may produce AO with various characteristics to better fit individual preferences.

## 2. Materials and methods

### 2.1. Reagents

6-hydroxy-2,5,7,8-tetramethylchromane-2-carboxylic acid (trolox), tocopherols (*α*-, *β*-, *γ-*, and *δ*-), phytosterols, squalene, fatty acid methyl esters (37 FAME mix), 2,2′-azinobis (3-ethylbenzothiazoline)-6-sulfonic acid (ABTS) and 1,1-diphenyl-2-picrylhydrazyl (DPPH) were obtained from Sigma-Aldrich (Bellefonte, PA, USA). All other solvents were of chromatography grade and ultrapure water (Milli-Q system, Millipore, Bedford, MA, USA) was used throughout the experiments.

### 2.2. Plant materials

Avocado fruits (*P. americana* Mill. cv. *Hass*, *Pinkerton*, and *Reed*) were harvested from Hainan, Chain in February 2020 and stored at room temperature until full maturity before manually separated into seeds, pulp and peel. The weight of the whole fruit, pits, peel and pulp of more than 50 avocado fruits were evaluated. The pulp was immersed into citric acid solution (1%, w/v) for 15 min to avoid possible enzymatic darkening (7).

### 2.3. Raw material pretreatment and oil extraction

Avocado pulp was pulped into puree in a beater with a creamy consistency and then either oven-dried or vacuum-dried or freeze-dried as detailed below. The puree was spread onto stainless steel trays (80 × 100 cm) and then either dried in an oven (Fanen 320 SE, Brazil) with forced air circulation at 60°C or in a vacuum drier (Memmert Inc. VO 500 model, Germany) at 40°C and 600 mbar for 24 h. The temperatures were chosen according to the results of dos Santos et al. (8). For freeze drying, pulp was lyophilized at-40°C for 24 h with a freeze-drier (Edwards Pirani 501, West Sussex, United Kingdom). All drying was performed in triplicate and the dried pulp was vacuum packaged with light-proof bags and stored at-50°C for further use. Oil extraction was performed by Soxhlet extraction with petroleum ether, as described by AOAC 920.39. Yield was calculated as the ratio of the weight of oil extract to the dry weight of puree.

### 2.4. Determination of physicochemical properties

The moisture content, dry weight, total sugars, crude fat, crude fiber, protein and ash contents were determined according to AOAC, 2000. Fat content was determined as the method described by Rodríguez-Carpena et al. (14). The slip melting point, iodine value, free fatty acids content, saponification value and unsaponifiable matter content were analyzed as described in AOCS, 1999.

### 2.5. Determination of fatty acid composition

The fatty acid composition was analyzed as the method stated by Tan et al. (15) with minor modifications. FAME solution was prepared by adding 0.1 g of oil into hexane solution (5 ml) and sodium methoxide reagent (250 μl). Then, 5 ml of sodium chloride solution (26.5%, w/v) was added. After vigorous shaking for 15 s, the solution was stood at room temperature for 10 min to allow stratification. The top layer (1 μl) was analyzed with a gas chromatograph (Thermo Fisher Scientific Inc., Waltham, MA, USA) coupled with a polar capillary column (30 m × 0.25 mm, 0.25 μm, Austin, Texas, USA) and a flame ionization detector. The initial column temperature was maintained at 100°C for 2 min and then increased to 230°C at a rate of 5°C/min and was held there for 10 min. The temperatures of the injector and the detector were fixed at 250°C throughout the analysis. FAME standards were employed to identify the peaks on the obtained chromatograms.

### 2.6. Determination of solid fat content

Solid fat content (SFC) was measured using a Bruker Minispec (Model Mq 20) pulse nuclear magnetic resonance (NMR) spectrometer (Karlsruhe, Germany). Samples in the NMR tubes were heated at 80°C for 60 min, and then held at the corresponding measuring temperatures. After 30 min, SFC measurements were taken at 5°C intervals over the range of 0–40°C.

### 2.7. Determination of nutritional components

The contents of tocopherols were determined by HPLC as previously described (16). Folin–Ciocalteu reagent was employed to determine the total phenolic content according to the method of Rubilar et al. (17) with some modification. Phytosterols and squalene were identified and quantified with GC–MS. (18)

### 2.8. Antioxidant activity

Antioxidant activity was assessed via DPPH assay as the method described by Sun et al. (19) with some changes. Briefly, 50 ml of various concentrations of AO solution (25, 50, 100, 200 or 250 mg/ml, dissolved in methanol) was blended with 2 ml of DPPH solution (24 mg/ml) and then incubated in the dark at room temperature. After 30 min, absorbance was then measured at 517 nm using a UV–visible spectrophotometer (Varian Cary 50, Mulgrave, Australia). Tert-butyl hydroquinone was recruited as a reference. Radical scavenging activity was calculated as follows: radical scavenging activity = (*A_DPPH −_ A_AO_*)/A_DPPH_ × 100%. *A_DPPH_* and *A_AO_* are the absorbance of the DPPH solution and the test solution, respectively.

### 2.9. Data analysis

All experiments were carried out in triplicate. Results are presented as mean ± standard deviations. Analysis of variance and multiple comparison was performed with Duncan’s tests. *p* values less than 0.05 were considered statistically significant.

## 3. Results and discussion

### 3.1. Morphometric characteristics and physical parameters of avocado fruits

An avocado fruit includes three anatomical parts: peel, pulp and seed. The morphometric measurement results of the whole fruit, peel, pulp and seed from the three avocado varieties are demonstrated in Table 1. In the light of all significantly different measurements among these avocado varieties, a clear differentiation among them was indicated. *Reed* showed the largest fruit and seed. *Hass* presented the lowest pulp percentage (60.59%), which agreed with the data reported by Costagli and Betti (20). Moisture content in the fresh pulp from *Hass* was 73.35 ± 0.45%, and its crude fat content was 16.53 ± 0.13%, which was significantly higher than that of *Pinkerton* (12.55 ± 1.43%) and *Reed* (14.25 ± 0.99%). This data was similar to a previous study that reported the lipids content in avocado pulp to range from 10 to 15%, showing its dependence on genotype as well as other factors (e.g., cultivar., season and growing conditions) (13, 21). Unlike oil extracted from other fruits such as palm kernel oil, AO is extracted from pulp rather than seeds as they contain very little oil (<2%) and have hepatoxic substances (22). Furthermore, compared to *Reed* and *Pinkerton* varieties, *Hass* variety is rich in edible oils (23). Therefore, the pulp of *Hass* would be a better candidate for obtaining AO.

**Table 1:** Physicochemical properties of the three avocado varieties (*Hass*, *Pinkerton*, *Reed*).

Physiochemical properties	Varieties		
	Hass	Pinkerton	Reed
whole fruit weight (g)	178.17 ± 14.52^aC^	240.28 ± 13.67^aB^	294.98 ± 4.56^aA^
Peel weight (g)	37.76 ± 4.10^cB^	48.17 ± 5.11^cA^	47.77 ± 9.24^cA^
Pulp weight (g)	108.24 ± 12.38^bC^	171.53 ± 13.95^bB^	211.49 ± 5.27^bA^
Seed weight (g)	32.17 ± 3.72^cA^	20.58 ± 2.55^dB^	35.72 ± 8.53^cA^
Pulp percentage (%)	60.59 ± 2.01^bB^	71.29 ± 1.75^aA^	71.69 ± 1.68^aA^
Humidity (%)	73.35 ± 0.45^bB^	80.25 ± 1.60^aA^	75.08 ± 2.00^bB^
Total dry extract (%)	26.65 ± 0.45^aA^	25.25 ± 0.41^aA^	23.56 ± 0.48^aA^
Total sugars (%)	0.18 ± 0.01^aA^	0.16 ± 0.02^aA^	0.14 ± 0.01^aA^
Crude fat (%)	16.53 ± 0.13^aA^	12.55 ± 1.43^bB^	14.25 ± 0.99^bB^
Proteins (%)	4.61 ± 0.02^aA^	2.20 ± 0.60^bC^	3.33 ± 0.21^bB^
Crude fiber (%)	5.02 ± 0.21^aA^	4.08 ± 0.51^aB^	5.42 ± 0.68^aA^
Ashes (%)	1.00 ± 0.03^aA^	0.95 ± 0.03^aB^	0.92 ± 0.08^aC^

^a^Values with different letters (a-b) within a column are significantly different (*p* < 0.05). Values with different letters (A-B) within a row are significantly different (*p* < 0.05). Experiments were carried out in triplicate. Results are presented as mean ± standard deviations.

### 3.2. Comparison of oil yields and quality indices of avocado oils

**Figure 1:**
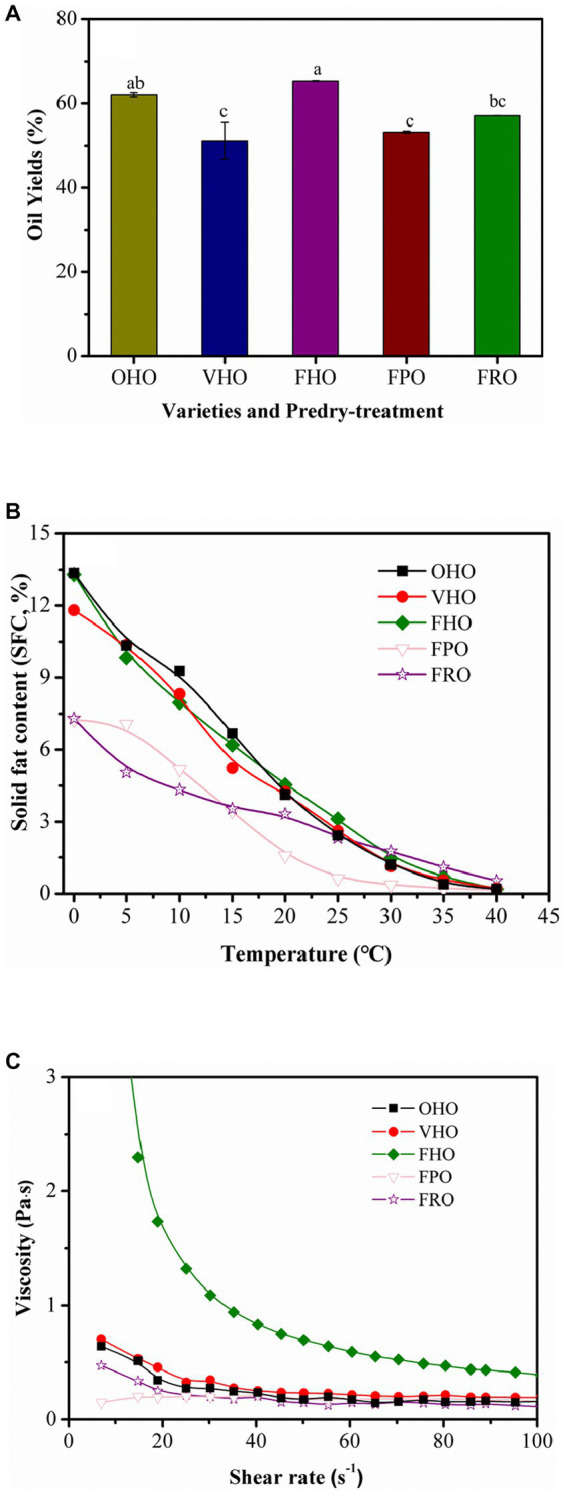
Oil yields **(A)**, solid fat content **(B)** and viscosity **(C)** of the AOs extracted from avocado pulp treated with different drying methods. OHO: oven-dried *Hass* oil, VHO: vacuum-dried *Hass* oil, FHO: freeze-dried *Hass* oil, FPO: freeze-dried *Pinkerton* oil, FRO: freeze-dried *Reed* oil. Experiments were carried out in triplicate. Results are presented as mean ± standard deviations.

AOs were extracted from the three avocado varieties that were subjected to different predry-treatments (freeze drying, oven drying and vacuum drying). The yields of the five oils are demonstrated in Figure 1A. It was found that drying methods had significant effect on AO yield with the highest yield obtained by freeze drying. Comparatively, vacuum drying pretreatment produced the lowest oil yield (51.13 ± 4.33%). This result was in accordance with other studies (2, 24). The changes of AO yield resulted from different drying treatments could result from drying temperature and drying method (9). Besides, the results also indicated that under the same kind of drying method (freeze drying) significant different AO yields could be observed from varieties (Figure 1A). The highest oil yield (65.30 ± 0.10%) was observed for *Hass* variety, which was 12.10 and 8.23% higher than those of *Pinkerton* and *Reed* varieties, respectively. Therefore, freeze drying treatment could increase oil yield and *Hass* showed the highest extraction efficiency among the three varieties investigated in the present study. This agreed well with some earlier works (7, 24).

Figure 1B shows the SFC profile of the AOs. The SFCs of freeze-dried *Hass* oil (FHO), freeze-dried *Pinkerton* oil (FPO) and freeze-dried *Reed* oil (FRO) at 0°C were 13.36, 7.30 and 7.25%, respectively, and decreased almost to 0% at 40°C, showing consistency to previous findings (25, 26). Notably, FHO stood out from the other two oils (FPO and FRO) due to its higher SFC in a wide temperature range. This is contrary to the results reported by Yanty et al. (27), who detected low SFC for Australian *Hass* oil. However, the *Hass* oil from West Malaysia displayed high SFC from 13.57 to 18.00% at 0°C. This difference could result from the variance in fatty acids and triglycerides composition (25). In terms of the influence of drying treatments, the SFC curves for oven-dried *Hass* oil (OHO), vacuum-dried *Hass* oil (VHO) and FHO intertwined with each other in a small range, suggesting no obvious difference was induced from drying treatments. Therefore, *Hass* can be a promising base material in making cream and lotion for skin nourishment. *Reed* and *Pinkerton*, on the other hand, could be used for making sauce, marinade and salad dressing. In general, these results illustrate that the variety but not predry-treatment affects the SFC in AO. With respect to the viscosity profile (Figure 1C), FHO is remarkably different from others, which may be due to its unique physicochemical properties (2, 28).

### 3.3. Physicochemical properties and bioactive constituents of the avocado oils

The two factors influencing the functionality of oil are physicochemical properties and bioactive constituents. Therefore, the appearance, microstructure, fatty acid composition, quality indices as well as the tocopherol, phytosterol, squalene and polyphenol contents of the AOs obtained from the three avocado varieties that were dried with different methods were examined systematically.

#### 3.3.1. Physicochemical properties of avocado oils

**Figure 2:**
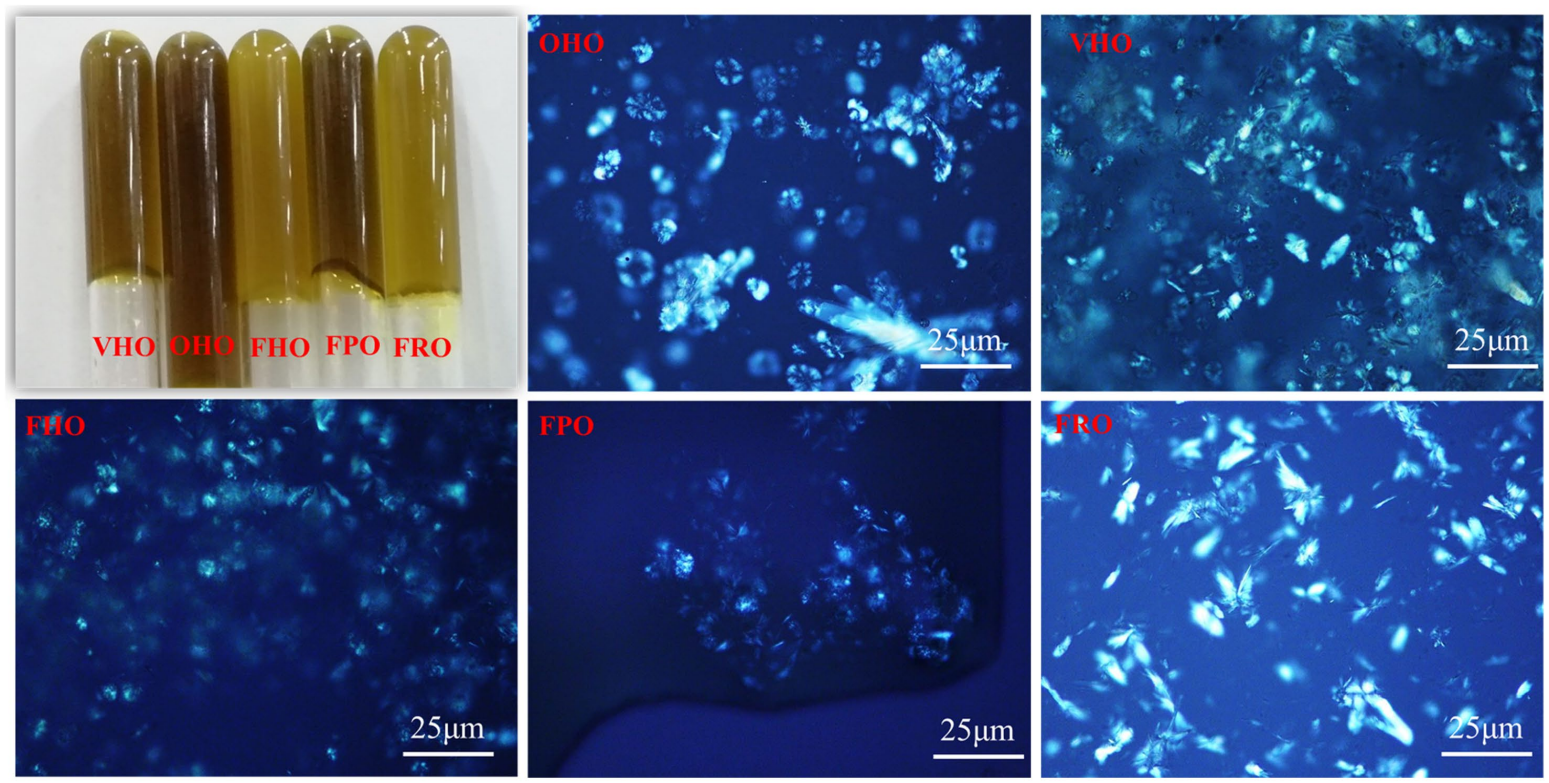
Appearance and polarized photomicrograph of the AOs extracted from avocado pulp treated with different drying methods. OHO: oven-dried *Hass* oil, VHO: vacuum-dried *Hass* oil, FHO: freeze-dried *Hass* oil, FPO: freeze-dried *Pinkerton* oil, FRO: freeze-dried *Reed* oil.

The AOs appeared olive drab or olive color (Figure 2), which was partly due to the existence of chlorophylls and carotenoids. Comparatively, the color of FHO was lighter than that of OHO and VHO, which might because lower temperature in lyophilization prevented polyphenols from oxidation (24). A homologous result was reported by Tan et al. (15) that described darker color in tray-dried AO. This indicated AO extracted by freeze-dried avocado pulp was lighter-colored. Moreover, FHO and FRO exhibited lighter color than FPO, alluding that the variety also has an influence on the oil appearance. The microstructure of these AOs was also investigated (Figure 2). For the oil from *Hass* avocado, spherulites resulted from the aggregation of needle-like crystals that grown from nucleation centers with several branches were observed. Meanwhile, sparse plate crystals were revealed in FRO and very few crystals were observed in FPO. These results show that there are obvious differences in the physical properties of the oils extracted from the three avocado varieties.

#### 3.3.2. Fatty acids profile in avocado oils

The fatty acid composition of the five AOs was determined and quantified by gas chromatography. As shown in Table 2, seven fatty acids were identified, and the content of palmitic, palmitoleic, oleic and linolenic acids differed obviously. Specifically, the predominant fatty acid was oleic acid (C18:1, n-9, *cis*), followed by palmitic (C16:0), linoleic (C18:2, n-6, *cis*) and palmitoleic (C16:1, n-7, *cis*) acids. Correspondingly, stearic (C18:0) and *α*-linolenic (C18:3, n-3, *cis*) acids were barely found, which agreed with the observation of Yanty et al. (27) who found that both stearic acid and *α*-linolenic acid contents were below 1%. The distribution of these fatty acids in the AOs from *Hass*, *Reed* and *Pinkerton* is homothetic to many past studies, such as that in palm oil and lard (29, 30). Lignoceric (24:0), arachidic (20:0) and *cis*-11-eicosenoic (20:1 n-9) were found to have a rather low content (< 0.3%), for which their results are not shown here. Interestingly, the content of the monounsaturated oleic acid (C18:1, from 33.25 to 39.10%) was lower than that in the oils from avocado harvested from other parts of the world (45.9–54.5%) (21, 31). This discordance could result from climate and geographical variations. In particular, monounsaturated palmitoleic acid (1, 16), with pharmaceutical applications in anti-inflammation and triglycerides-lowering effect, was the most abundant fatty acid (9–14%). The major saturated fatty acid (palmitic acid), with the content ranging from 26 to 29%, was the second most abundant fatty acid. These fatty acid contents in the five AOs were as expected for avocado and showed general consistency with a previous report on avocado fruit (11, 32). The fatty acid profiles of the five oils by different predry-treatments were very similar (Table 2). However, the *Hass* variety had higher abundance of palmitoleic acid than many other varieties (3–10%) (33), which reflected the potential of avocado as a dietary supplement to help lower blood cholesterol level and prevent cardiovascular diseases as well as dermatosis. Besides, a high UFAs/SFAs ratio (>2.0) is considered beneficial to human health; the higher the ratio, the higher the nutritional quality of a vegetable oil (34).

**Table 2:** Fatty acid composition of the five AOs extracted from avocado pulp treated with different predry-treatments.^*^

Fatty acid profiles	OHO	VHO	FHO	FPO	FRO
Palmitic acid C16:0	26.13 ± 0.04	29.63 ± 0.21	28.04 ± 0.06	28.79 ± 0.07	29.02 ± 0.09
Palmitoleic acid C16:1 (n-7)	13.03 ± 0.08	13.17 ± 0.10	14.53 ± 0.05	9.77 ± 0.07	14.24 ± 0.05
Stearic acid C18:0	0.62 ± 0.01	0.67 ± 0.02	0.67 ± 0.01	0.86 ± 0.00	0.69 ± 0.01
Oleic acid C18:1 (n-9)	28.02 ± 0.06	29.66 ± 0.08	29.81 ± 0.12	34.93 ± 0.30	30.44 ± 0.02
Oleic acid C18:1 (n-7)	5.23 ± 0.10	5.62 ± 0.04	6.23 ± 0.20	4.18 ± 0.21	6.07 ± 0.02
Oleic acid C18:1	33.25 ± 0.92	35.35 ± 0.09	36.05 ± 0.30	39.10 ± 0.50	36.52 ± 0.03
Linoleic acid C18:2	15.79 ± 0.06	17.04 ± 0.11	16.61 ± 0.05	16.29 ± 0.05	16.49 ± 0.04
Linolenic acid C18:3	0.70 ± 0.01	1.16 ± 0.02	0.76 ± 0.01	0.95 ± 0.00	0.71 ± 0.01
SFAs	26.75 ± 0.08	30.30 ± 0.21	28.71 ± 0.23	29.65 ± 0.10	29.71 ± 0.09
MUFAs	46.28 ± 0.89	48.52 ± 0.09	50.58 ± 0.28	48.87 ± 0.67	50.76 ± 0.08
PUFAs	62.77	66.72	67.95	66.11	67.95
UFAs/SFAs	2.35	2.20	2.36	2.23	2.29

^*^MUFAs: monounsaturated fatty acids; PUFAs: polyunsaturated fatty acids; SFAs: saturated fatty acids. UFAs: unsaturated fatty acids (= MUFAs + PUFAs); OHO: oven-dried *Hass* oil, VHO: vacuum-dried *Hass* oil, FHO: freeze-dried *Hass* oil, FPO: freeze-dried *Pinkerton* oil, FRO: freeze-dried *Reed* oil. Experiments were carried out in triplicate. Results are presented as mean ± standard deviations.

#### 3.3.3. Peroxide value, anisidine value, and acid value

**Figure 3:**
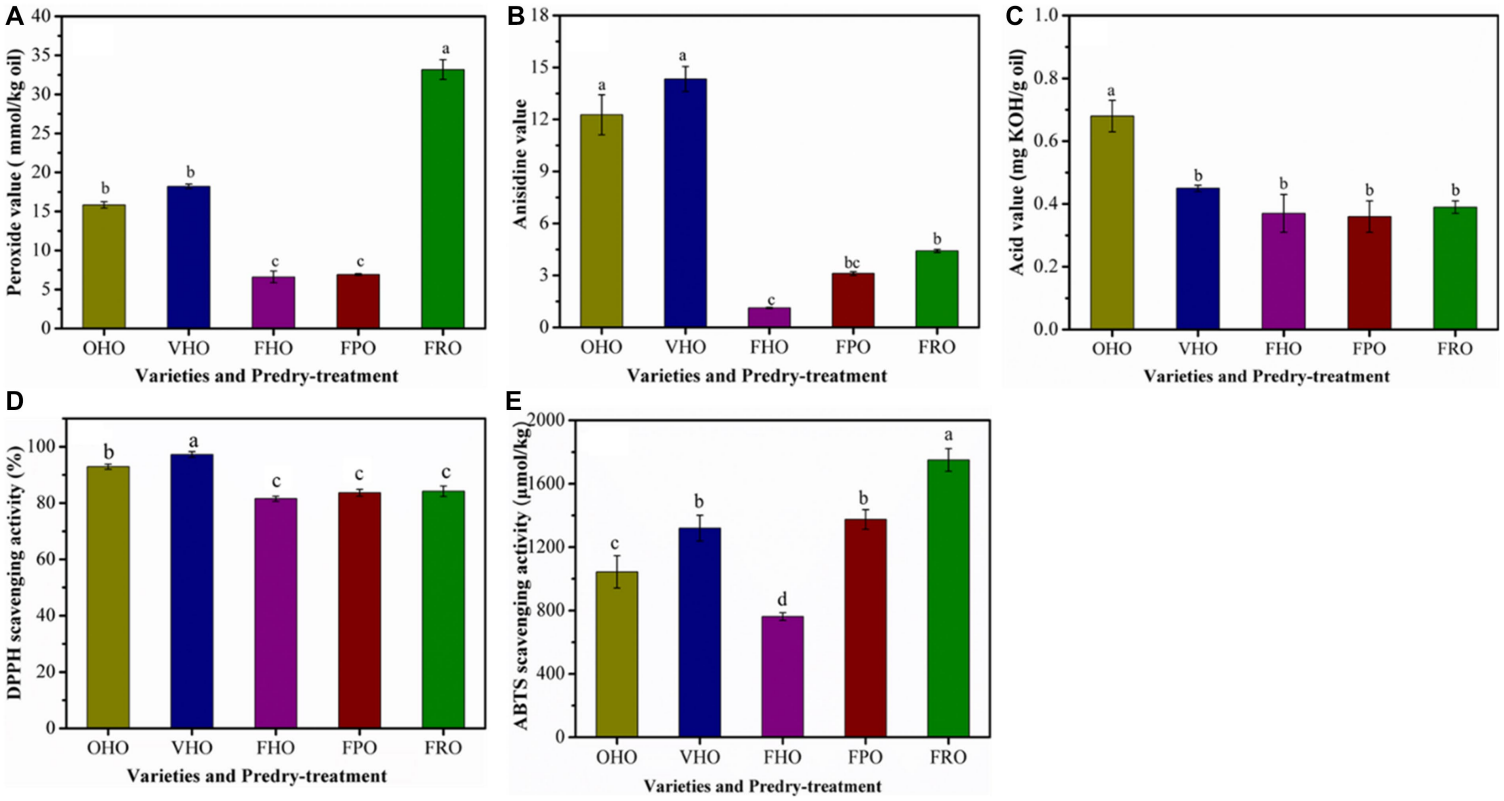
Peroxide value **(A)**, anisidine value **(B)**, acid value **(C)**, DPPH radical scavenging activity **(D)** and ABTS radical scavenging activity **(E)** of the AOs extracted from avocado pulp treated with different drying methods. OHO: oven-dried *Hass* oil, VHO: vacuum-dried *Hass* oil, FHO: freeze-dried *Hass* oil, FPO: freeze-dried *Pinkerton* oil, FRO: freeze-dried *Reed* oil. Experiments were carried out in triplicate. Results are presented as mean ± standard deviations.

The predry-treatment had significantly influenced the peroxide, anisidine and acid value of the AOs. The main products of primary degradation of lipids autoxidation are peroxides. FHO presented the lowest peroxide value than other oils (Figure 3A), which could originate from the low temperature in freeze drying that provides considerable protection against lipid oxidation. This result suggests that the peroxide value of AO is dependent on the predry-treatment. Moreover, under the same predry-treatment, peroxide value of AO varied significantly with avocado varieties, where FRO showed the highest peroxide value. The anisidine content stands for the degree of secondary degradation of lipids. Correspondingly, the anisidine value of FHO was also significantly lower than other oils (Figure 3B). The free fatty acid content indicates the presence of hydrolytic degradation that is associated with off flavor and oil changes (35). The acid value of OHO was significantly lower than that of VHO and FHO (Figure 3C), suggesting that the free fatty acid content in AO was affected by predry-treatment. Further, FHO showed the lowest acidity level (0.37 mg KOH/g). Even so, the acidity level of *Hass* oil either by oven or vacuum drying pretreatment and *Pinkerton* as well as *Reed* oil exceeded the threshold of 4 mg KOH/g oil, which abided by the international standards of crude, cold-pressed oil and indicated that there was no considerable hydrolysis or oxidation of lipids during pulp processing and oil extraction. This agreed with the results of Krumreich et al. (7). Taken together, all the findings purported that the AO extracted from *Hass* by freeze drying was of good quality.

#### 3.3.4. Bioactive compounds content

AO is rich in bioactive compounds that can serve as antioxidants and improve human health. The contents of bioactive components in the five AOs are shown in Table 3. *α*-Tocopherol appeared as the dominant tocopherol in all AOs. In terms of the effect of drying techniques, oils from freeze-dried pulps (FHO) showed the highest contents of tocopherols. The main sterols in the AOs were campesterol, Δ5-avenasterol, *β*-sitosterol and stigmasterol, where *β*-sitosterol was the most predominant one. Variety, planting location and different test methods could induce the high variation in total sterols content. The results also revealed significant variation in the total phenolic content of the AOs with respect to both predry-treatment and variety. With respect to the unsaponifiable fraction, squalene was the major component (205.58–611.27 mg/kg), whose content was higher that of total tocopherols and phenolics. FRO had higher squalene content (611.27 mg/kg) than FPO (306.15 mg/kg) and FHO (218.93 mg/kg). These AOs present much greater squalene content in comparison to that in the oils from Brazilian, Mexican and Australian avocados (3, 28).

**Table 3:** Tocopherols, phytosterols, polyphenols and squalene contents of AOs extracted from avocado pulp treated with different predry-treatments.^*^

Components	OHO	VHO	FHO	FPO	FRO
Tocopherols (mg/kg)					
*α*-Tocopherol	109.08 ± 0.78^c^	94.45 ± 0.01^d^	129.71 ± 2.70^b^	124.56 ± 3.01^b^	194.39 ± 3.59^a^
*α*-Tocotrienols	28.66 ± 8.26^a^	24.973 ± 2.76^a^	22.19 ± 1.11^a^	33.17 ± 9.77^a^	28.43 ± 3.65^a^
*δ*-Tocotrienols	2.72 ± 0.14^a^	2.959 ± 0.08^a^	2.70 ± 0.05^a^	2.81 ± 0.29^a^	3.29 ± 0.18^a^
Total	140.46 ± 9.18b^c^	122.379 ± 2.82^c^	154.61 ± 1.54^b^	160.54 ± 13.16^b^	226.12 ± 7.42^a^
Phytosterols (mg/g)					
Campesterol	0.30 ± 0.01^c^	0.33 ± 0.01^b^	0.32 ± 0.01^bc^	0.418 ± 0.01^a^	N.D.^#^
Δ5-Avenasterol	0.04 ± 0.01^a^	0.38 ± 0.01^a^	0.05 ± 0.00^a^	0.045 ± 0.01^a^	N.D.^#^
*β*-Sitosterol	3.68 ± 0.03^b^	3.85 ± 0.00^b^	3.56 ± 0.08^b^	4.288 ± 0.17^a^	N.D.^#^
Stigmasterol	0.15 ± 0.00^a^	0.16 ± 0.02^a^	0.18 ± 0.01^a^	0.191 ± 0.02^a^	N.D.^#^
Total	4.16 ± 0.03^b^	4.38 ± 0.01^b^	4.10 ± 0.08^b^	4.942 ± 0.20^a^	N.D.^#^
Phenolic (mg/g)	24.66 ± 0.17^b^	31.49 ± 0.19^a^	29.64 ± 0.56^c^	23.28 ± 2.31^bc^	33.63 ± 0.19^a^
Squalene (mg/kg)	205.58 ± 0.36^c^	241.52 ± 0.16^c^	218.93 ± 0.10^c^	306.15 ± 0.21^b^	611.27 ± 31.27^a^

^*^OHO: oven-dried *Hass* oil, VHO: vacuum-dried *Hass* oil, FHO: freeze-dried *Hass* oil, FPO: freeze-dried *Pinkerton* oil, FRO: freeze-dried *Reed* oil. Values with a different letter within a row are significantly different (*p <* 0.05).

^#^N.D.: not detected. Experiments were carried out in triplicate. Results are presented as mean ± standard deviations.

### 3.4. Antioxidant activity of the avocado oils

The antioxidant capacities of the AOs determined by DPPH and ABTS assay are presented in Figure 3. The DPPH scavenging capacity ranged from 81 to 97%, which indicated favorable radical scavenging activity of all the AOs (Figure 3D). In terms of the effect of predry-treatments, vacuum drying endowed AO with higher radical scavenging activity as DPPH scavenging activity of VHO was greater than that of OHO and FHO. In addition, *Hass* oil extracted from oven-dried and vacuum-dried avocado pulp exhibited significantly higher DPPH scavenging capability than that of *Pinkerton* and *Reed* ones. With respect to ABTS scavenging activity, the *Reed* oil exhibited the highest capacity, followed by *Pinkerton* oil and *Hass* oil (Figure 3E). The high ABTS scavenging activity of *Pinkerton* oil was probably associated with the content of tocopherols and phenolic (Table 3). Similar to the results obtained in the DPPH assays the *Hass* oil from vacuum-dried pulp showed higher radical scavenging activity than that of oils from other predry-treatments. Likewise, a great decrease in the antioxidant activity of phenolic compounds was observed after its subjection to oven-drying (14, 23, 36).

## 4. Conclusion

The changes in the bioactive constituents and quality parameters of AOs from three Chinese avocado varieties subjected to different predry-treatments (oven drying, vacuum drying and freeze drying) was investigated in this study. It was found that both avocado varieties and drying methods had significant influence on the oil composition and oil quality. The best extraction yield of AO was obtained by the combination of freeze drying and *Hass* variety. More importantly, it was also found that AO extracted from freeze-dried *Hass* pulp has high nutritional quality, as suggested by its fatty acid composition, peroxide value, anisidine value, acid value, bioactive compounds profile and antioxidant activity. The findings of the present study are important to improve the quality of Chinese AO and shed light on the future exploitation of *Hass* variety as a raw material to produce functional oils that can be applied to food or cosmetic industry.

## Data availability statement

The original contributions presented in the study are included in the article/supplementary material, further inquiries can be directed to the corresponding authors.

## Author contributions

JW: conceptualization, methodology, writing – original draft, writing – review and editing, and funding acquisition. HY: formal analysis, investigation, visualization, and writing – original draft. PW, JZ, WM, YL, and JL: conceptualization, supervision, and writing – review and editing. All authors contributed to the article and approved the submitted version.

## Funding

This work was financially supported by Key R&D Projects of Hainan Province (ZDYF2021XDNY162) and Central Public-interest Scientific Institution Basal Research Fund for Chinese Academy of Tropical Agricultural Sciences (No. 16300920210010).

## Conflict of interest

The authors declare that the research was conducted in the absence of any commercial or financial relationships that could be construed as a potential conflict of interest.

## Publisher’s note

All claims expressed in this article are solely those of the authors and do not necessarily represent those of their affiliated organizations, or those of the publisher, the editors and the reviewers. Any product that may be evaluated in this article, or claim that may be made by its manufacturer, is not guaranteed or endorsed by the publisher.
